# Low expression of polypeptide GalNAc *N*-acetylgalactosaminyl transferase-3 in lung adenocarcinoma: impact on poor prognosis and early recurrence

**DOI:** 10.1038/sj.bjc.6601531

**Published:** 2004-01-20

**Authors:** C Gu, T Oyama, T Osaki, J Li, M Takenoyama, H Izumi, K Sugio, K Kohno, K Yasumoto

**Affiliations:** 1 Department of Thoracic and Cardiovascular Surgery, First Affiliated Hospital of Dalian Medical University, Dalian 116011, China; 2 Department of Surgery II University of Occupational and Environmental Health, School of Medicine, 1-1 Iseigoaka, Yahatanishi-ku Kitakyushu 807-8555, Japan; 3 Department of Molecular Biology, University of Occupational and Environmental Health, School of Medicine, 1-1 Iseigoaka, Yahatanishi-ku Kitakyushu 807-8555, Japan

**Keywords:** GalNAc-T3, lung adenocarcinoma, prognosis, recurrence

## Abstract

Initial glycosylation of mucin-type O-linked protein is catalysed by one of the UDP-GalNAc: polypeptide *N*-acetyl-galactosaminyl transferase-3 (GalNAc-T3). O-glycosylation is important in the binding of cell adhesion molecules, cell differentiation, invasion, and metastasis in tumours. This study was designed to detect GalNAc-T3 expression in lung adenocarcinoma by using immunohistochemical staining, and to evaluate the relationship between the GalNAc-T3 expression level and prognosis and recurrence in completely resected lung adenocarcinoma patients. A low expression of GalNAc-T3 was detected in the cytoplasm of tumour cells in 79 of 148 patients (53.4%) with lung adenocarcinoma. The low expression of GalNAc-T3 was associated with poorly differentiated tumour (*P*<0.0001), poor pathologic stage (*P*<0.0001), lymph node metastasis (*P*<0.0001), and tumour recurrence (*P*=0.016). The lung carcinoma patients with low GalNAc-T3 expression had a poorer prognosis than those with high GalNAc-T3 expression, using both univariate and multivariate analyses (overall survival: *P*<0.0001 and *P*=0.011, respectively). In addition, multivariate analysis of the clinicopathological characteristics of stage I lung adenocarcinoma indicated that the low expression of GalNAc-T3 was a significant independent factor for predicting poor prognosis and early recurrence (*P*=0.006, rr=2.87 and *P*=0.019, rr=3.05, respectively). The low expression of GalNAc-T3 may be a useful marker for predicting poor prognosis and early recurrence in completely resected lung carcinoma patients, particularly patients with stage I diseases.

It is widely accepted that changes in the structure and distribution of cell surface glycoproteins are assumed to influence the biologic behaviour of tumour cells during malignant transformation and tumour progression (Nakamori *et al*, 1994). In the past few years, O-linked carbohydrate antigens, such as mucin antigen (MUC1), carcinoembryonic antigen (CEA), sialyl LewisX (SLX), CA19-9, Sialyl Tn (STn), and Tn have been reported to be associated with altered adhesion (Fogel *et al*, 1983; Rice and Bevilacqua, 1989), invasion (Bolscher *et al*, 1988; Matsushita *et al*, 1991; Nakamori *et al*, 1994), recurrence, and prognosis (Itzkowitz *et al*, 1990; Niklinski *et al*, 1992; Ogawa *et al*, 1994; Cao *et al*, 1995; Diez *et al*., 1996; Ohgami *et al*, 1999) in lung cancer and other tumours. Mucin-type O-linked carbohydrates may constitute up to 80% of the total mass of these glycoproteins (Clausen and Bennett, 1996); O-glycosylation has been shown to be important in the binding of cell adhesion molecules, cell differentiation, invasion, and metastasis in tumours (Nakamori *et al*, 1994; Clausen and Bennett, 1996; Sutherlin *et al*, 1997; Nomoto *et al*, 1999). Initial glycosylation of mucin-type O-linked protein was catalysed by a family of the UDP-GalNAc: polypeptide *N*-acetyl-galactosaminyl transferases (GalNAc-transferase) (Hagen *et al*, 1993; Homa *et al*, 1993; Wandall *et al*, 1997). To date, seven distinct human GalNAc-transferase genes (from GalNAc-T1 to GalNAc-T7) have been cloned and characterised, and O-glycosylation is carried out in part by the differential expression of GalNAc-transferase in normal tissues and tumours (White *et al*, 1995; Bennett *et al*, 1996, 1998, 1999a, 1999b; Wandall *et al*, 1997; Ten Hagen *et al*, 1998). Thus, the differential expression of O-linked carbohydrate antigens in tumours may be explained by the differential expression and activity of specific GalNAc-transferases.

GalNAc-T3 expression is restricted to cell lines derived from epithelial gland adenocarcinoma, but not cells derived from nonsecretory epithelial tissue carcinomas (Sutherlin *et al*, 1997; Nomoto *et al*, 1999). A recent study using a polyclonal antibody to GalNAc-T3 demonstrated that GalNAc-T3 is expressed in adenocarcinoma but not nonadenocarcinoma cell lines (Nomoto *et al*., 1999). Previous immunohistochemical results suggest that the anti-GalNAc-T3 antibody may be useful for diagnostic purposes in the early stages of breast cancer, but the relationship between GalNAc-T3 expression and prognosis and recurrence for patients with lung adenocarcinoma remains unknown.

This study was a retrospective cohort and designed to detect GalNAc-T3 expression in lung adenocarcinoma by using immunohistochemical staining, and to evaluate the relationship between GalNAc-T3 expression levels and prognosis or recurrence of the patients' tumours.

## MATERIALS AND METHODS

### Patients and follow-up

We examined 148 of 184 (80.4%) consecutive patients with lung adenocarcinoma, who underwent complete surgical resection between July 1991 and September 1996 at the University of Occupational and Environmental Health, School of Medicine, Kitakyushu, Japan. The criteria for inclusion into the study were based on the availability of follow-up data. Clinicopathological data were obtained by retrospective chart review. Tumour stage was classified according to Revisions in the International System for Staging Lung Cancer (1997) (Mountain, 1997). There were 88 men and 60 women in this series, with a mean age of 64.8 years (range, 32–84). None of these patients received chemotherapy or radiotherapy prior to the operation. Of 148 (6.8%) patients, 10 received adjuvant chemotherapy and this adjuvant chemotherapy does not affect the prognosis of these patients.

For the postoperative follow-up, the patients were examined every month within the first year and at approximately 2- to 4-month intervals thereafter. The evaluations included physical examination, chest roentgenography, analysis of blood chemistry, and carcinoembryonic antigen assay. Chest, abdominal, and brain computed tomographic scans and a bone scintiscan were performed every 6 months through the third year, and annually thereafter. If any symptoms or signs of recurrence appeared in these examinations, additional evaluations to locate the site of the recurrence were performed. Survival data were updated in April 2001. Follow-up was available for all patients, ranging from 53 to 3507 days after the primary operation (median follow-up, 48.5 months).

### Western blot analysis

Cytoplasmic proteins were extracted from the frozen normal tissue and tumour tissue in adenocarcinoma patients and 5 *μ*g cytoplasmic proteins was electroblotted onto polyvinylidene difluoride membranes (Immobilon; Millipore, Bedford, MA, USA) after separation on 10% SDS–polyacrylamide gel electrophoresis gel. Polyclonal antibody against GalNAc-T3 was generated by multiple immunisations of a New Zealand white rabbit, using synthetic peptides as described previously (Nomoto *et al*, 1999). Immunoblot analysis was performed with a 1 : 5000 dilution of anti-GalNAc-T3 antibody. Detection was performed using ECL (Amersham Pharmacia Biotech, Buckinghamshire, UK). High GalNAc-T3 expression breast carcinoma given by Nomoto *et al* (1999) was used as a positive control.

### Immunohistochemical staining

A formalin-fixed, paraffin-embedded, 3-*μ*m section was obtained from each of the 148 samples of primary lesions. All specimens were stained with haematoxylin and eosin for histopathologic diagnosis. Immunohistochemical (IHC) staining was performed by a streptavidin–biotin–peroxidase complex method (Nomoto *et al*, 1999). Sections were immersed briefly in a citrate buffer (0.01 mol li^−1^ citric acid: pH 6.0) and incubated for two 5-min intervals at 100°C in a microwave oven for antigen retrieval. They were then incubated with polyclonal GalNAc-T3 antibody diluted 1 : 2000 for 90 min at room temperature by using the Labeled Streptavidin Biotin kit (DAKO LSAB kit, CA930 13, Dako Corp., Carpinteria, CA, USA). Antibody was diluted in phosphate-buffered saline (PBS) containing 2% bovine serum albumin (BSA). High GalNAc-T3 expression breast carcinoma given by Nomoto *et al* (1999) was also used as a positive control.

### Immunostaining evaluation

All slides were evaluated for immunostaining by three observers (CG, JL, and TO) using a blind protocol design (observers had no information on clinical outcome or other clinicopathologic data). Cells were judged positive for GalNAc-T3 when the cytoplasm or cell membranes were stained. The percentage of positive cells was calculated by counting more than 1000 cells in random high-power fields (10 × 40), and scored according to the percentage of positive GalNAc-T3 cells: score 0, 0–5%; score 1, 6–25%; score 2, 26–50%; score 3, 51–75%; or score 4, 76–100% expression levels. To evaluate the correlation with clinicopathological characteristics, GalNAc-T3 expression scores were divided into two groups. Specimens with expression scores of 0–2 were called low expression of GalNAc-T3, and specimens with scores of 3–4 were called high expression of GalNAc-T3.

### Statistical analysis

The statistical significance was evaluated using the Pearson's *χ*^2^ test. Survival curves were plotted according to the Kaplan–Meier method (Kaplan and Meier, 1958), and differences between the curves were analysed by the log-rank test (Peto *et al*, 1977). The Cox proportional hazards model was applied to the multivariate survival analysis (Cox, 1972). The results of the Cox proportional hazards model did not seem to change when the follow-up was within 5 years. Data were analysed with the use of Abacus Concepts, Survival Tools for StatView (Abacus Concepts, Inc., Berkeley, CA, USA).

## RESULTS

### Western blot analysis

**Figure 1:**
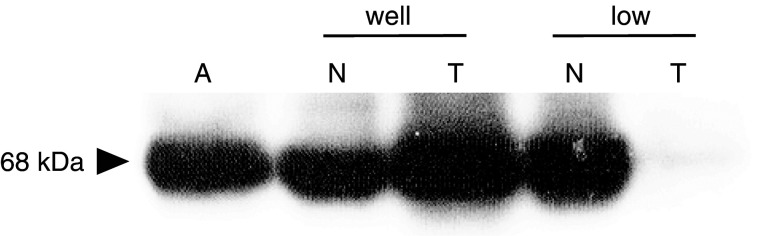
Western blotting analysis showed GalNAc-T3 expression levels of normal tissue and tumour tissue of patients with well-differentiated adenocarcinoma and poorly differentiated adenocarcinoma. Lane A; positive control (extracted from high GalNAc-T3 expression breast carcinoma), N; normal lung tissue, T; tumour tissue, well; well-differentiated adenocarcinoma, poorly; poorly differentiated adenocarcinoma.

To determine the specificity of an anti-GalNAc-T3 antibody, Western blot analysis was performed in 10 cases with frozen normal tissue and tumour tissue from the same patient. GalNAc-T3 expression was detected in 68 kDa protein. High GalNAc-T3 expression breast carcinoma given by Nomoto *et al* (1999) was used as a positive control (lane A). Figure 1

shows the one of Western blotting analysis, in which samples were extracted from normal tissue and tumour tissue of patients with well-differentiated adenocarcinoma and poorly differentiated adenocarcinoma. GalNAc-T3 expression level of tumour tissue increased in comparison with that of normal tissue in well-differentiated adenocarcinoma, although the GalNAc-T3 expression level of tumour tissue decreased in comparison with that of normal tissue in poorly differentiated adenocarcinoma. The concordance rate between the results of Western blot analysis and the results of IHC detection about GalNAc-T3 expression was 80%.

### Immunohistochemical detection of GalNAc-T3 expression in lung adenocarcinoma

**Figure 2:**
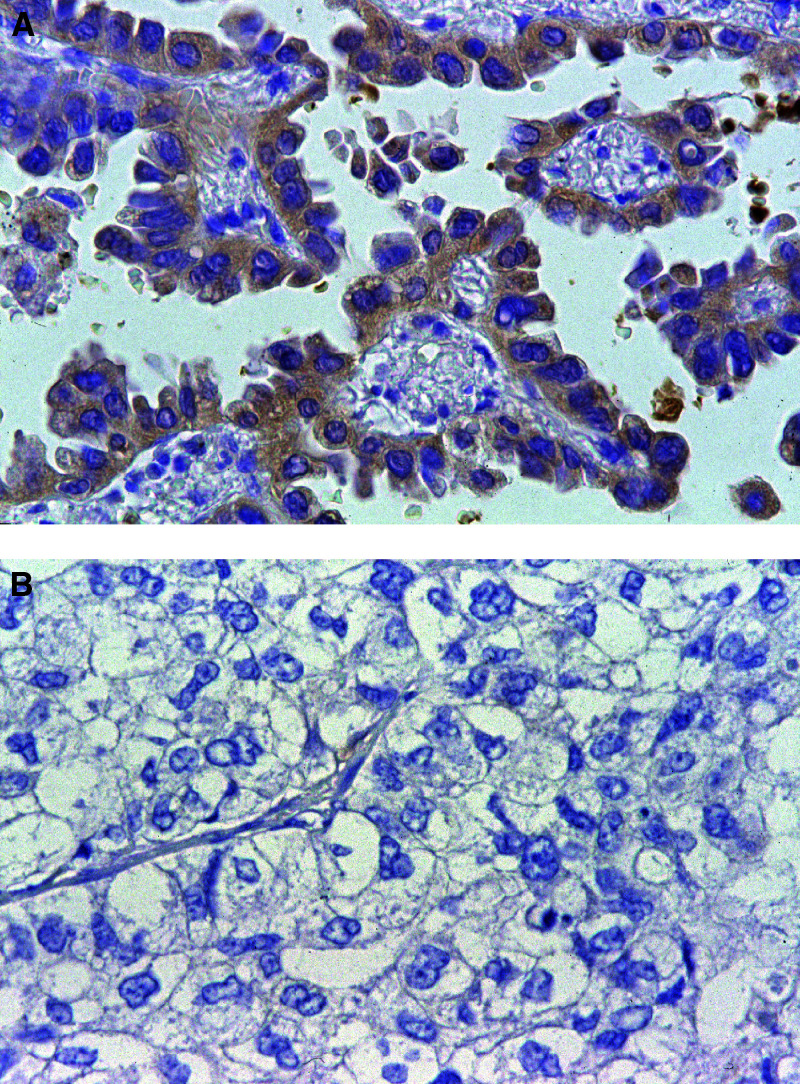
GalNAc-T3 IHC detection in adenocarcinoma. (**A**) High GalNAc-T3 expression tumour cells with deep brown stained cytoplasm are shown in the case of well-differentiated adenocarcinoma (original magnification × 400). (**B**) Low GalNAc-T3 expression tumour cells without stained cytoplasm are shown in the case of poorly differentiated adenocarcinoma (original magnification × 400).

In all 148 specimens, 69 (46.6%) stained positive for GalNAc-T3 in the cytoplasm of over 50% of tumour cells, and 79 (53.4%) showed a low expression of GalNAc-T3 in the cytoplasm. In tumour cells, the GalNAc-T3 IHC staining was usually seen in the cytoplasm or cell membranes. In a few cases, immunostaining was also observed in the nucleus as well as in a chromosome in mitosis. But the surrounding normal stromal cells did not react. In normal lung tissues, GalNAc-T3 IHC staining was often seen in the respiratory epithelium and bronchial glands. Typical appearances of staining in high expression of GalNAc-T3 and low expression of GalNAc-T3 tumours are shown in Figure 2a and b

, respectively. The relations between GalNAc-T3 expression level and various clinicopathologic characteristics of the patients are summarised in Table 1

**Table 1:** Relations between the level of GalNAC-T3 expression and clinicopathologic characteristics in 148 lung adenocarcinoma

		**GalNAC-T3**			
**Characteristics**	**Total**	**Low**	**(%)**	**High**	***P***
All cases	148	79	(53.4)	69	
*Gender*					
Male	88	54	(61.4)	34	
Female	60	25	(41.7)	35	0.02
*Age (years)*					
<65	68	42	(61.8)	26	
≥65	80	37	(46.3)	43	0.06
*Histologic Grade*					
Well	44	12	(27.3)	32	
Moderately	74	38	(51.4)	36	
Poorly	30	29	(96.7)	1	<0.01
*Pathologic stage*					
1	78	28	(35.9)	50	
2	9	4	(44.5)	5	
3	53	42	(79.2)	11	
4	8	5	(62.5)	3	<0.01
*pTNM*					
T					
T1	56	24	(42.9)	32	
T2	51	26	(51.0)	25	
T3	15	10	(66.7)	5	
T4	26	19	(73.1)	7	0.05
*N*					
N0	91	33	(36.3)	58	
N1	14	10	(71.4)	4	
N2	41	34	(82.9)	7	
N3	2	2	(100)	0	<0.01
*M*					
M0	140	74	(52.9)	66	
M1	8	5	(62.5)	3	0.60
*Recurrence*					
Without recurrence	106	50	(47.2)	56	
With recurrence	42	29	(69.1)	13	0.02

. The frequency of low GalNAc-T3 expression was significantly lower in well-differentiated adenocarcinomas (27.3%) than in those with moderately and poorly differentiated adenocarcinomas (51.4 and 96.7%, respectively) (*P*<0.01). Thus, GalNAc-T3 expression in tumours showed a significant relationship to histologic differentiation. As shown in Table 1, low expression of GalNAc-T3 was associated with pathologic stage (pStage) (*P*<0.01), lymph node metastasis (*P*<0.01), and tumour recurrence (*P*=0.016), but not distant metastasis (*P*=0.60). A borderline significant *P*-value was also found in T status (*P*=0.05).

Next, GalNAc-T3 expression level was compared to various clinicopathologic parameters of patients with stage I lung adenocarcinoma (tumours with no invasion of adjacent structures and no metastases to regional lymph nodes or distant organs). As shown in Table 2

**Table 2:** Relations between the level of GalNAC-T3 expression and clinicopathologic characteristics in stage I adenocarcinoma

		**GalNAC-T3**			
**Characteristics**	***n***	**Low**	**(%)**	**High**	***P***
All cases	78	28	(35.9)	50	
*Histologic grade*					
Well	31	6	(19.4)	25	
Moderately	38	14	(36.8)	24	
Poorly	9	8	(88.9)	1	<0.01
*Pathologic stage*					
1A	46	15	(32.6)	31	
1B	32	13	(40.6)	19	0.47
*Recurrence*					
Without recurrence	57	16	(28.1)	41	
With recurrence	21	12	(57.1)	9	0.02

, among 78 patients with lung adnocarcinoma in stage I, a significant relationship was observed between GalNAc-T3 expression and histologic differentiation. In addition, a low expression of GalNAc-T3 was detected in 12 out of 21 (57.1%) cases with recurrent disease, and this finding was statistically significant (*P*=0.02). Of the 12 patients, 10 experienced early tumour recurrence (within 2 years of the primary operation). Finally, the pattern of recurrence seemed to be haematogenous. In all, 10 patients had haematogenous distant recurrences, one patient had a lymphogenous recurrence, and one patient had haematogenous recurrences in combination with lymphogenous metastases. Thus, the low expression of GalNAc-T3 showed a significant relationship to early tumour recurrence.

### Influence of GalNAc-T3 expression level on survival and recurrence

**Figure 3:**
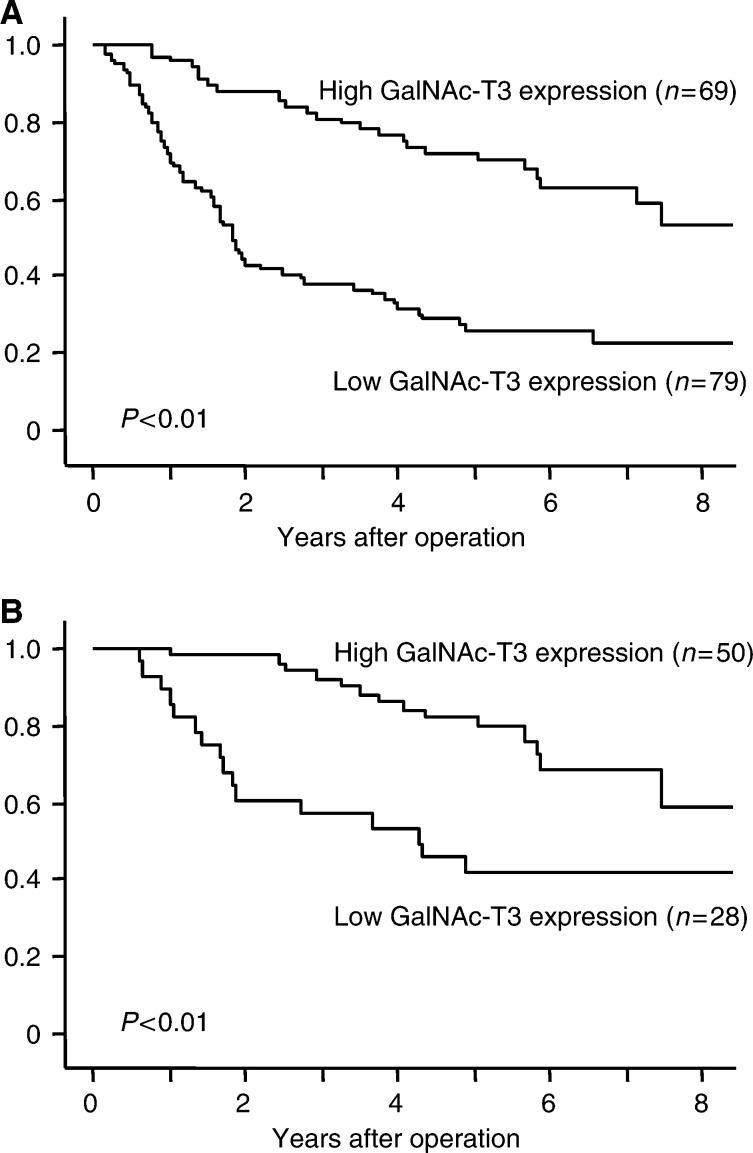
Survival curves of adenocarcinoma patients with low GalNAc-T3 expression or high GalNAc-T3 expression. (**A**) Overall survival (*n*=148), the overall 5-year survival rates in the patients with low GalNAc-T3 expression and high GalNAc-T3 expression were 25.4 and 72.1%, respectively (*P*<0.01). (**B**) Survival curves of stage I lung adenocarcinoma patients (*n*=78, *P*<0.01).

The influence of GalNAc-T3 expression level on the patients' overall survival was evaluated. The overall 5-year survival rate was 47.4% for the 148 patients in this study. The Kaplan–Meier survival curves demonstrated that the patients with low GalNAc-T3 expression had significantly shorter survival periods than those with high GalNAc-T3 expression (*P*<0.01) (Figure 3A

). The patients with low GalNAc-T3 expression (score 0 and 1) also had significantly shorter survival periods than those with high GalNAc-T3 expression (score 4) (*P*<0.01, data not shown) when the survival curves of three groups patients were determined, according to the cutoff points of 25 and 75%. Among the patients with stage I (*n*=78), the overall 5-year survival rates in the patients with low GalNAc-T3 expression and high GalNAc-T3 expression were 41.6 and 82%, respectively (*P*<0.01) (Figure 3B). In univariate analysis, six variables (gender, pT, pN, pM, grade of differentiation, and high or low expression of GalNAc-T3) were found significantly to affect survival in 148 adenocarcinoma patients. Furthermore, a multivariate survival analysis demonstrated that five variables (pT, pN, pM, grade of differentiation, and high or low expression of GalNAc-T3) were independently associated with patient survival (Table 3

**Table 3:** Univariate and multivariate analyses using a proportional hazard model for survival of 148 lung adenocarcinoma patients

		**Univariate analysis**			**Multivariate analysis**		
**Variable**	***n***	**95% CI**	**HR**	***P***	**95% CI**	**HR**	***P***
*Gender*							
Female	60		1			1	
Male	88	1.02–2.54	1.61	0.04	0.71–1.85	1.15	0.58
*pT*							
T1	56		1			1	
T2	51	1.39–4.45	2.49	<0.01	1.28–4.38	2.37	<0.01
T3	15	1.96–8.52	4.09	<0.01	1.06–5.37	2.38	0.04
T4	26	3.54–12.5	6.66	<0.01	2.16–8.72	4.34	<0.01
*PN*							
N0	91		1			1	
N1	14	1.86–6.94	3.59	<0.01	0.76–3.18	1.55	0.23
N2	41	2.31–5.98	3.71	<0.01	1.05–3.20	1.83	0.03
N3	2	1.23–21.7	5.17	0.03	2.46–59.1	12.1	<0.01
*pM*							
M0	140		1			1	
M1	8	1.85–9.26	4.13	<0.01	0.93–5.18	2.19	0.07
*Grade*							
Well	44		1			1	
Moderately	74	2.09–7.66	4.00	<0.01	1.46–6.07	2.98	<0.01
Poorly	30	2.11–9.37	4.44	<0.01	0.74–3.88	1.69	0.22
GalNAC-T3							
High expression	69		1			1	
Low expression	79	2.20–5.65	3.53	<0.01	1.26–3.91	2.22	<0.01

HR-hazard ratio.

). The low expression of GalNAc-T3 was estimated to be associated with an increased risk of death by a factor of 2.22 in multivariate analysis (*P*<0.01). On the other hand, in univariate analysis, three variables (pStage, grade of differentiation, and high or low expression of GalNAc-T3) were found to affect significantly survival in 78 patients with stage I adenocarcinoma. Furthermore, a multivariate survival analysis demonstrated that two variables (grade of differentiation, and high or low expression of GalNAc-T3) were independently associated with patient survival and low expression of GalNAc-T3 was estimated to be associated with an increased risk of death by a factor of 2.99 in multivariate analysis (*P*<0.01; data not shown).

**Figure 4:**
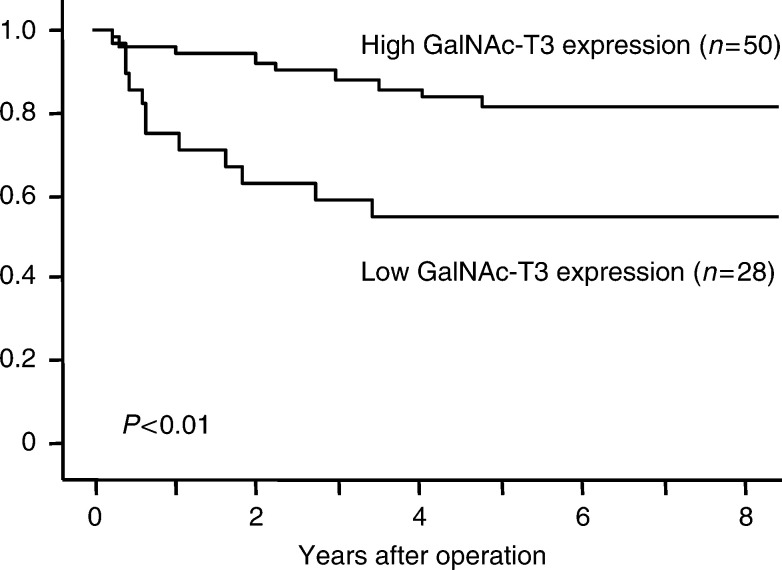
Disease-free survival curves of stage I lung adenocarcinoma patients with low GalNAc-T3 expression or high GalNAc-T3 expression (*n*=78). The disease-free 3-year survival rates in the patients with low GalNAc-T3 expression and high GalNAc-T3 expression were 59 and 87.9%, respectively (*P*<0.01).

To investigate whether GalNAc-T3 expression level is significantly correlated with recurrence in early-stage diseases, disease-free survival curves of the patients with GalNAc-T3 expression in stage I were analysed using the Kaplan–Meier method. As shown in Figure 4

, the patients with low GalNAc-T3 expression had significantly shorter disease-free survival periods than those with high GalNAc-T3 expression (*P*<0.01). In univariate analysis, two variables (grade of differentiation and high or low expression of GalNAc-T3) were found to affect significantly disease-free survival in 78 patients with stage I adenocarcinoma. The variable of pStage was estimated as a borderline association (*P*=0.06). A multivariate diseade-free survival analysis demonstrated that the variable of grade of high or low expression of GalNAc-T3 was independently associated with disease-free survival. The low expression of GalNAc-T3 was estimated to be associated with an increased risk of death by a factor of 3.05 in multivariate analysis (*P*=0.02) (Table 4

**Table 4:** Univariate and multivariate analyses using a proportional hazard model for disease-free survival of 78 patients with stage I adenocarcinoma

		**Univariate analysis**			**Multivariate analysis**		
**Variable**	**n**	**95% CI**	**HR**	***P***	**95% CI**	**HR**	***P***
*pStage*							
1A	46		1			1	
1B	32	0.96–5.43	2.29	0.06	0.83–5.01	2.04	0.12
Grade							
Well	31		1			1	
Moderately	38	1.12–10.3	3.40	0.03	0.87–8.39	2.70	0.09
Poorly	9	0.79–16.0	3.56	0.10	0.25–6.60	1.29	0.76
*GalNAC-T3*							
High expression	50		1			1	
Low expression	28	1.38–7.86	3.29	<0.01	1.22–7.65	3.05	0.02

HR=hazard ratio.

).

## DISCUSSION

Lung cancer is the leading cause of cancer-related deaths in North America, and it became the most common cause of cancer-related deaths among Japanese in 1998 (Ginsberg *et al*, 1997; Hara *et al*, 2000). Of the four main histologic types of lung cancer, adenocarcinoma is the most commonly occurring type in Japan (Hara *et al*, 2000). Lung cancer is also an aggressive carcinoma with a poor outcome, and the overall 5-year survival rate is about 20% (Landis *et al*, 1998; Hara *et al*, 2000). Therefore, it is important to evaluate the malignant potential of tumour cells for a more precise evaluation of the prognosis for patients with lung adenocarcinoma.

The current study investigated the associations between GalNAc-T3 expression level and various clinicopathologic characteristics of patients with lung adenocarcinoma. The low expression of GalNAc-T3 was associated with poorly differentiated tumour, poorly pathologic stage, lymph node metastasis and tumour recurrence. Both univariate and multivariate analyses demonstrated that, among the clinicopathologic factors examined, low expression of GalNAc-T3 was a significant independent factor for predicting poor prognosis and early recurrence.

Concerning the differentiation status of lung adenocarcinoma and GalNAc-T3 expression levels, low expression levels of GalNAc-T3 correlated with poorly differentiated adenocarcinoma and high expression levels of GalNAc-T3 correlated with well-differentiated adenocarcinoma. These findings are consistent with the report by Sutherlin *et al* (1997) that GalNAc-T3 expression is higher in well-differentiated pancreatic adenocarcinoma cell lines. Generally, poorly differentiated adenocarcinoma are thought to have a greater malignant potential than well-differentiated adenocarcinoma. Thus, GalNAc-T3 expression level may be a marker of malignant potential in lung adenocarcinoma.

It is well known that the metastatic process consists of several stages: tumour cell escape from primary tumour, invasion of the vessels, migration, adhesion to the vascular endothelium, extravasation, and colonisation, all of which are essential for the development of clinically overt metastases. Among O-linked carbohydrate antigens, sialyl LewisX has been reported to function as a ligand of the endothelial cell adhesion molecule E-selectin (ELAM-1), which adheres human cancer cells to the vascular endothelium (Phillips *et al*, 1990; Walz *et al*, 1990; Takada *et al*, 1993). In addition, a mucin antigen such as MUC1 inhibits the E-cadherin-mediated cell–cell adhesion, which implies that MUC1 mucin enables adenocarcinoma cells to be detached readily from the primary tumour tissue (Wesseling *et al*, 1996). MUC1 polypeptide is also associated with cell surface O-linked glycoproteins such as sialyl LewisX and sialyl LewisA (Ho *et al*, 1995). The initial glycosylation of mucin-type O-linked carbohydrate antigens is regulated in part by the differential expression of GalNAc-transferase, which add GalNAc to fibronectine (Bennett *et al*. 1999b) and may affect the density or positions of attachment of O-linked oligosaccharides (Sutherlin *et al*, 1997). Differential expression of GalNAc-transferase may have a ‘valvular’ function in regulating O-glycosylation in mucin-type O-linked carbohydrate antigens such as sialyl LewisX and MUC1 (Wandall *et al*, 1997; von Mensdorff-Pouilly *et al*, 2000). Therefore, GalNAc-transferase expression may play a role in tumour cell escape from primary tumour, adhesion to the vascular endothelium, and extravasation during the metastatic process.

In lung cancer, even after a ‘curative’ resection for pathologic stage I disease, about 30% of patients experience recurrence and eventually die of the disease (Little *et al*, 1986; Ginsberg *et al*, 1997). This suggests that occult metastases are present at the time of surgical intervention. Previous studies (Gu *et al* 2002) showed that micrometastatic tumour cells (cytokeratin positive cells) were present in pathologic negative lymph nodes in 34.7% of stage I non-small-cell lung cancer (NSCLC) patients after complete resectioning, and patients with micrometastatic tumour cells had a poor prognosis and a high rate of recurrent disease. To see whether GalNAc-T3 expression correlates with micrometastases, micrometastatic tumour cells were detected in a total of 1436 hilar and mediastinal pathologic negative lymph nodes from 65 patients with stage I lung adenocarcinoma, using the method previously described (Gu *et al*, 2002), Of 65 patients, 19 exhibited lymph nodal micrometastasis, and a low expression of GalNAc-T3 was found in 12 patients (63.2%) (*P*=0.003). Furthermore, this may be a partial explanation for the relationship between aberrant expression of GalNAc-T3 and early metastasis (unpublished data, with subjects different from those of this study). In the current study, among 12 out of 78 patients with low GalNAc-T3 expression and recurrent diseases in stage I lung adenocarcinoma, tumour recurrence within 2 years of the primary operation was found in 10 patients. The pattern of recurrence seemed to be haematogenous, and a low expression of GalNAc-T3 was associated with poor prognosis and early recurrence. Based on these results, it is reasonable to argue that a low expression of GalNAc-T3 may be a useful indicator of early tumour recurrence in stage I lung adenocarcinoma.

In conclusion, low expression of GalNAc-T3 may be a useful marker in predicting poor prognosis and early recurrence, not only in all completely resected patients with adenocarcinoma of the lung but also in those with stage I diseases. These patients need to be followed up carefully after surgery. At present, postoperative adjuvant chemotherapy is not a routine standard therapy for completely resected NSCLC patients, because it has not been shown to improve patient outcomes consistently. However, by assessing the GalNAc-T3 expression level, it would be possible to select patients who might benefit most from adjuvant chemotherapy.
